# Levels and Patterns of Objectively Assessed Physical Activity and Compliance with Different Public Health Guidelines in University Students

**DOI:** 10.1371/journal.pone.0141977

**Published:** 2015-11-04

**Authors:** Natalia María Arias-Palencia, Monserrat Solera-Martínez, Luis Gracia-Marco, Pedro Silva, Vicente Martínez-Vizcaíno, Jorge Cañete-García-Prieto, Mairena Sánchez-López

**Affiliations:** 1 Universidad de Castilla-La Mancha, Health and Social Research Centre, Castilla-La Mancha, Cuenca, Spain; 2 Universidad de Castilla-La Mancha, Faculty of Education, Castilla-La Mancha, Cuenca, Spain; 3 Universidad de Castilla-La Mancha, Faculty of Occupational Therapy, Speech Therapy and Nursing, Castilla-La Mancha, Cuenca, Spain; 4 University of Exeter, CHERC Sport and Health Sciences, Exeter, United Kingdom; 5 Universidad de Zaragoza, GENUD “Growth, Exercise, Nutrition and Development” Research Group, Zaragoza, Spain; 6 University of Porto, Faculty of Sport, Research Centre in Physical Activity, Health and Leisure, Porto, Portugal; 7 Facultad de Ciencias de la Salud, Universidad Autónoma de Chile, Talca, Chile; 8 Universidad de Castilla-La Mancha, Faculty of Education, Castilla-La Mancha, Ciudad Real, Spain; University of the Balearic Islands, SPAIN

## Abstract

**Background:**

Physical activity (PA) is associated with health enhancement. The aim of this study was to assess: 1) levels and patterns of PA in university students by using accelerometers; and 2) the percentage of fulfilment of PA recommendations for adults, according to different public health guidelines.

**Methods:**

Observational cross-sectional study (Cuenca’s Adults Study) involving 296 (206 women) healthy Spanish university students aged 18–25 years old. Participants wore the ActiGraph GT1M accelerometer for seven consecutive days. Total PA, steps and time spent in sedentary time, light, moderate, vigorous, and moderate to vigorous PA (MVPA) was assessed, and the prevalence of sufficient PA was calculated according to various public health guidelines.

**Results:**

No sex differences in total PA were found. University students were more sedentary during weekend days than weekdays (p<0.05). Only 30.3% of participants accumulated 30 min/day at least five days a week of MVPA. A total of 5.4% of students met the recommendation of 150 min/week of MVPA or 75 min/week of vigorous PA, in PA bouts of at least 10 min. using the same definition, but on five or more days a week, only 0.5% students were found to meet the recommendation. In addition, only 0.5% of students met the recommendation of 30 min/day of MVPA, at least five days a week and in bouts of at least 10 min. Finally, 28.1% of the students met the recommendation of 10,000 steps/day.

**Conclusions:**

Our study shows a high incidence of sedentary time in university students. The number of students meeting PA recommendations significantly differed depending on the recommendation proposed. Specific strategies to promote PA in this population are necessary as well as an agreement as to which PA guidelines should be used.

## Background

Consistent evidence shows that regular physical activity (PA) helps to maintain good health levels and prevents diseases such as hypertension, obesity, metabolic syndrome, heart disease, diabetes, osteoporosis and some cancers, and produces significant benefits at psychosocial level [1,2]. However, despite its benefits, a high percentage of adults in most developed countries do not meet the current recommendations for PA, and their PA level is very low [3– 6].

It is known that the levels of PA decrease from childhood to adolescence, as well as from adolescence to adulthood [7,8]. Adolescence is a period in which physiological and psychological changes take place; whereas, the transition to young adulthood implies important lifestyle changes. Young adults spend more time in sedentary behaviours, and consume large amounts of alcohol and tobacco during the university period [8,9], leading to an unhealthy lifestyle and an increased cardiovascular risk [10].

Several studies have objectively measured the levels and PA patterns in young adults [4–6,11–15]. However, college students’ PA has been measured subjectively and inconsistently, which makes comparisons of PA patterns among different samples very difficult [16,17]. In Spanish university students, there is a lack of data of objectively measured PA behaviours. Since these PA habits are modifiable, and the benefits of changes in this behaviour are greater the earlier they occur, identifying PA patterns will enable us to design programmes for PA in order to prevent future health problems.

Several PA public health guidelines for healthy adult populations have been published over the last years; however, they differ in the amount, intensity and frequency of PA. In 1995, the *Centers for Disease Control and Prevention (CDC) and the American College of Sports Medicine (ACSM)* [18] recommended that ‘every adult should accumulate 30 minutes or more of moderate-intensity PA on most, preferably all days of the week’. In 2007, the *American Heart Association (AHA)* and *ACSM* [19] issued a revised recommendation clarifying that ‘moderate PA had to be done for a minimum of 30 min on five days/week and/or 20 min vigorous PA on three days/week, allowing the combination of both, and in episodes of at least 10 min’. In 2008, the *US Department of Health and Human Services* [20] and in 2010, the *World Health Organization* (WHO) [21] recommended at least 150 min of moderate aerobic PA/week or 75 min of vigorous aerobic PA/week, also allowing the combination of the two intensities, and in episodes of at least 10 min. The *British Association of Sport and Exercise Sciences (BASES)* [22] added that this aerobic PA had to be done on five or more days/week. Concurrently, PA public health guidelines have also been launched taking into account the number of steps/day with 10,000 steps/day as a reference [23]. This disparity in the recommendations on PA implies that the degree of compliance increases or decreases depending on the public health guidelines taken as a reference, and as a consequence the estimates of association with health outcomes also differ.

Several studies have reported the level of adult adherence to different PA public health guidelines [5,12,24–27]. Some have compared the compliance to different public health guidelines in the same population [4,15,28–30], but to our knowledge there are no similar studies of Spanish university students. Therefore, the aims of this study were to assess in a cross-sectional study in Spanish university students: 1) levels and patterns of PA using an objective measure, and 2) the percentage of fulfilment of PA recommendations for adults, according to different public health guidelines.

## Materials and Methods

### Study Design and Population

The Cuenca Adults Study is a longitudinal study aimed at assessing the changes in lifestyle and cardiovascular risk factors that occur during an individual’s time at university [31–33].

**Fig 1:**
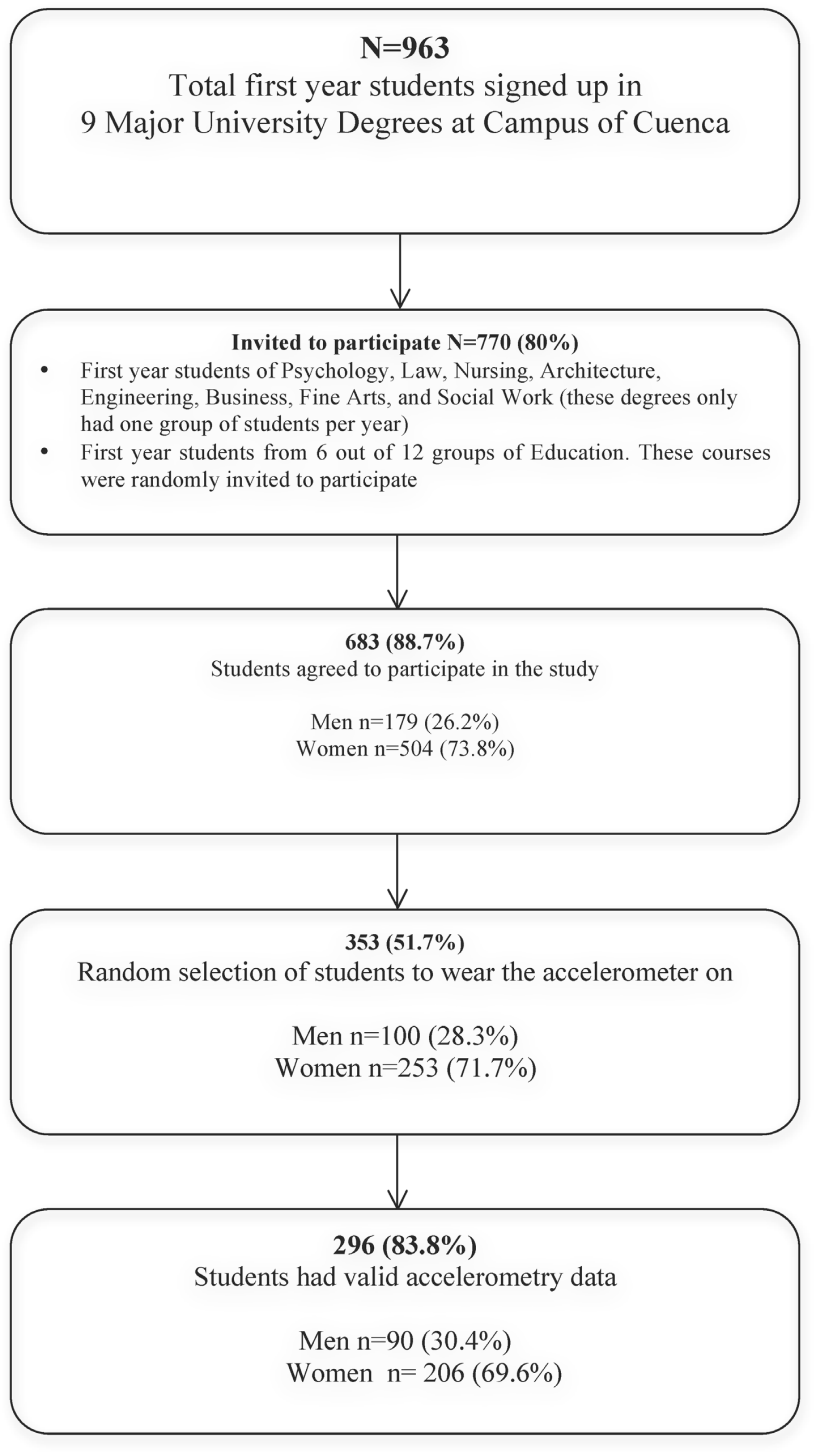
Flowchart showing the progress of students through the study.

The measurements of this study were conducted in 2009–2010 in the Cuenca campus (University of Castilla-La Mancha, Spain). From a total of 963 students in their first year, 770 students were invited to participate. They were all first year students of eight different degrees (Psychology, Law, Nursing, Architecture, Engineering, Business, Fine Arts and Social Work) and only 6 of 12 groups of first year of Education grade which were randomly invited to participate (Fig 1). Only the Nursing degree course was related to health. From the 770 invited students, 683 (88.7%) agreed to participate (504 woman, 73.8%). This sex distribution was similar to the whole campus student population (4,814 students, 63% women, n = 3,022), so that some degrees such as Nursing or Social Work could be mainly preferred by women.

The investigators attended first year courses to explain the aims of the study and invite participants; those interested signed the informed consent.

Students received a T-shirt and one European Credit Transfer System (ECTS.) for having participated in the study.

The university population included students older than 17 years old. Most of them lived in rented flats (40%) or in campus hostels (30%) close to the campus, so they are not far away from the lecturing places. In comparison to the other Castilla-La Mancha campuses, the campus of Cuenca is not very crowded and is located about a mile and a half away from the town centre. A sports service that organizes competitions and other recreational activities is situated in the campus. However, the student participation is quite poor. Only 3% of university women and 51% of men were engaged in competition activities and 19% of women and 23% of men in leisure activities.

The study protocol was approved by the Clinical Research Ethics Committee of Virgen de la Luz Hospital in Cuenca, and all subjects signed informed consent forms prior to their participation in the study.

### Anthropometry

The measurements were made in the University by trained investigators to minimize inter-observer variability. Body mass and height were measured following standardized recommendations with an electronic scale (SECA Model 861; Vogel & Halke, Hamburg, Germany, precision = 100 g, range = 0–150 kg), and a stadiometer (Type Seca 222, precision = 0.1 cm, range = 6–230 cm). The average of two measurements was taken. Body mass index (BMI) was calculated as body mass (in kilograms) divided by height^2^ (in metres).

### Assessment of physical activity

Participants wore an accelerometer (model MTI/CSA 7164, Actigraph, Shalimar, FL, USA) for seven consecutive days. A minimum of four days with valid data (minimum 600 registered min/day and including at least one weekend day) were set as inclusion criteria [33,34]. The device was worn on the right hip of the participants, attached with an elastic band. Because the accelerometers were not water-resistant, the subjects were asked to remove them before showering or entering a pool. In this study, the interval of recorded time (epoch) was set at 60 seconds, shown as valid for measuring PA in adults [35], and any 10-min intervals of continuous ‘zero’ activity were excluded from the analysis. Data were collected throughout the school year (September to July). Considering the students’ answers in the PA log (mean bedtime and wake up) it was considered ‘Awake time’ from 8.00 to 0.00 h. Accelerometer data were processed (Kinesoft v3.3.55) to provide values for each hour between 8.00 and 0.00 h for total daily and hourly counts per minute (cpm), sedentary time, light PA, moderate PA, vigorous PA, moderate-vigorous PA (MVPA), steps and non-wear time for weekdays and weekend. The intensity of weekly PA was assessed as average cpm. The cut-off values used to define the intensity of PA and, therefore, quantify the mean time in each intensity were the following: sedentary time = <100 cpm, light PA = 100–1951 cpm, moderate PA = 1952–5724, vigorous PA = ≥5725, and MVPA was calculated as the sum of moderate and vigorous PA [36].

Finally, the student percentage that had met different PA recommendations for adults was quantified and assessed. According to different public health guidelines, the sum of the min/day of moderate and vigorous PA and number of steps/day were processed. This was structured from low to high demand criteria of the guidelines [18–23] and by calculating the MVPA and vigorous PA in bouts of at least 10 minutes (allowing a minute or two below level marking):

- Recommendation 1 (R1): ≥30 min of MVPA for most of the week (5 days) [18].
- Recommendation 2 (R2): ≥150 min/week MVPA (R2A) and/or ≥75 min/week vigorous PA in bouts of at least 10 min (R2B) [20,21].
- Recommendation 3 (R3): ≥150 min/week MVPA (R3A) and/or ≥75 min/week vigorous PA (R3B) in bouts of at least 10 min in 5 or more days/week [22].
- Recommendation 4 (R4): ≥30 min/day MVPA × 5 days/week (R4A) and/or ≥20 min/day vigorous PA × 3 days/week (R4B) in bouts of at least 10 min [19].
- Recommendation 5 (R5): 10,000 steps/day [23].

The students who had less than five valid days were excluded from the analysis of recommendations R3A, R3B and R4A.

### Calculation of sample size

The sample size (n = 451) was calculated by means of the software StatsDirect estimating a prevalence of overweight/obesity of 25%, a confidence level of 95% and a precision of 4%. Estimating a rate of not response of 10%, the total size of the sample was 496 students.

### Statistical Analyses

The variables were checked for normal distribution both through graphical procedures and by the Kolmogorov-Smirnov test. Descriptive data were assessed by the t-test for normally distributed variables and Mann-Whitney’s U for non-normally distributed variables.

As none of the PA-related variables had a normal distribution, the Wilcoxon test was used to compare PA intensities on weekdays and weekends. Since an interaction between sex and the studied variables was not observed (all p>0.05), all analyses were performed for men and women together.

All statistical analyses were performed with the statistical software IBM^®^ SPSS^®^ Statistics 19, and the level of significance was set at p<0.05.

## Results

Fig 1 shows the progress of students through the study. From the 683 students who agreed to participate, a randomly selected subsample of 353 participants (51.7%, 253 women), aged 18–25 years old, was selected to wear the accelerometer. The final sample included 296 (83.8%, 206 women) first year university students with four days of valid accelerometry data (minimum 600 registered min/day and including at least one weekend day).

Anthropometric and PA characteristics are displayed in Table 1. There were no significant differences (p< 0.05) between men and women in PA-related variables. Overall, university students were more active on weekdays than weekend days, except for light PA and number of steps in males.

**Table 1:** Descriptive characteristics of the studied participants.

	Overall	Men	Women	P value
	(n = 296)	(n = 90)	(n = 206)	
Age (years)^a^	19.3±1.76	19.72±1.79	19.16±1.73	0.011
Height (m)	1.65(1.59–1.72)	1.74(1.70–1.80)	1.63(1.58–1.67)	< 0.001
Weight (Kg)	60.6(52.9–69.2)	71.1(62.9–79.9)	56.5(50.9–64)	< 0.001
BMI (Kg/m^2^)	21.8(19.9–24.1)	23.1(21.1–25.2)	21.3(19.7–23.4)	< 0.001
Total activity (cpm)	294.2(227.7–357.9)	281.3(224–360.2)	297.6(231.4–357.3)	0.565
Registered time (min/d)	822 ± 111.41	820.9 ± 104.62	822.4 ± 114.46	0.918
Valid days (no.)	5.26 ± 0.97	5.27 ± 0.92	5.26 ± 0.99	0.929
Non- wear time (min/d)	618 ± 111.37	619 ± 104.61	617.5 ± 114.4	0.918
*Weekday PA*				
Sedentary time (min/day)	595.2(540.1–647.2)*	601.3(547.1–664)*	593.4(534.6–641.8)*	0.213
Light PA (min/day)	253.3(217.5–287.2)*	256.3(223.35–284.26)	250.9(216–289.2)*	0.757
Moderate PA (min/day)	36.1(22.4–53.8)*	32.2(23–50.1)*	38.1(22–54.3)*	0.430
Vigorous PA (min/day)	0.33(0–1.57)*	0.33(0–2.05)*	0.25(0–1.5)*	0.318
MVPA	39.4(23.2–55)*	34.4(23.2–53.2)*	40.6(22.9–55.6)*	0.491
Steps^b^	9081(7128–10861)*	8994(7706–10949)	9132(6976–10811)*	0.445
*Weekend PA*				
Sedentary time (min/day)	618(542.5–689.7)	622.8(541.4–695.4)	617.8(549.6–688.5)	0.485
Light PA (min/day)	256.3(221.4–300.2)	251.65(220.4–300.4)	259.1(221.8–300.6)	0.890
Moderate PA (min/day)	22.3(10.5–40.5)	22.3(11.4–34.6)	22.3(9.9–43.1)	0.876
Vigorous PA (min/day)	0(0–0)	0(0–0)	0(0–0)	0.903
MVPA (min/day)	22.5(10.5–41.4)	22.8(11.4–37.1)	22.5(10.4–43.5)	0.858
Steps^b^	7971(5763–10430)	8439(6740–10516)	7656(5406–10399)	0.120

^a^Age is shown as mean (standard deviation) and descriptive anthropometric characteristics, physical activity (PA) characteristics and steps are shown as medians and interquartile range.

Abbreviations: PA, physical activity; BMI, body mass index; cpm, counts per minute; MVPA, moderate-to-vigorous physical activity.

*Significant differences between weekdays and weekend.

^b^Overall (n = 197), men (n = 62), women (n = 135).

**Fig 2:**
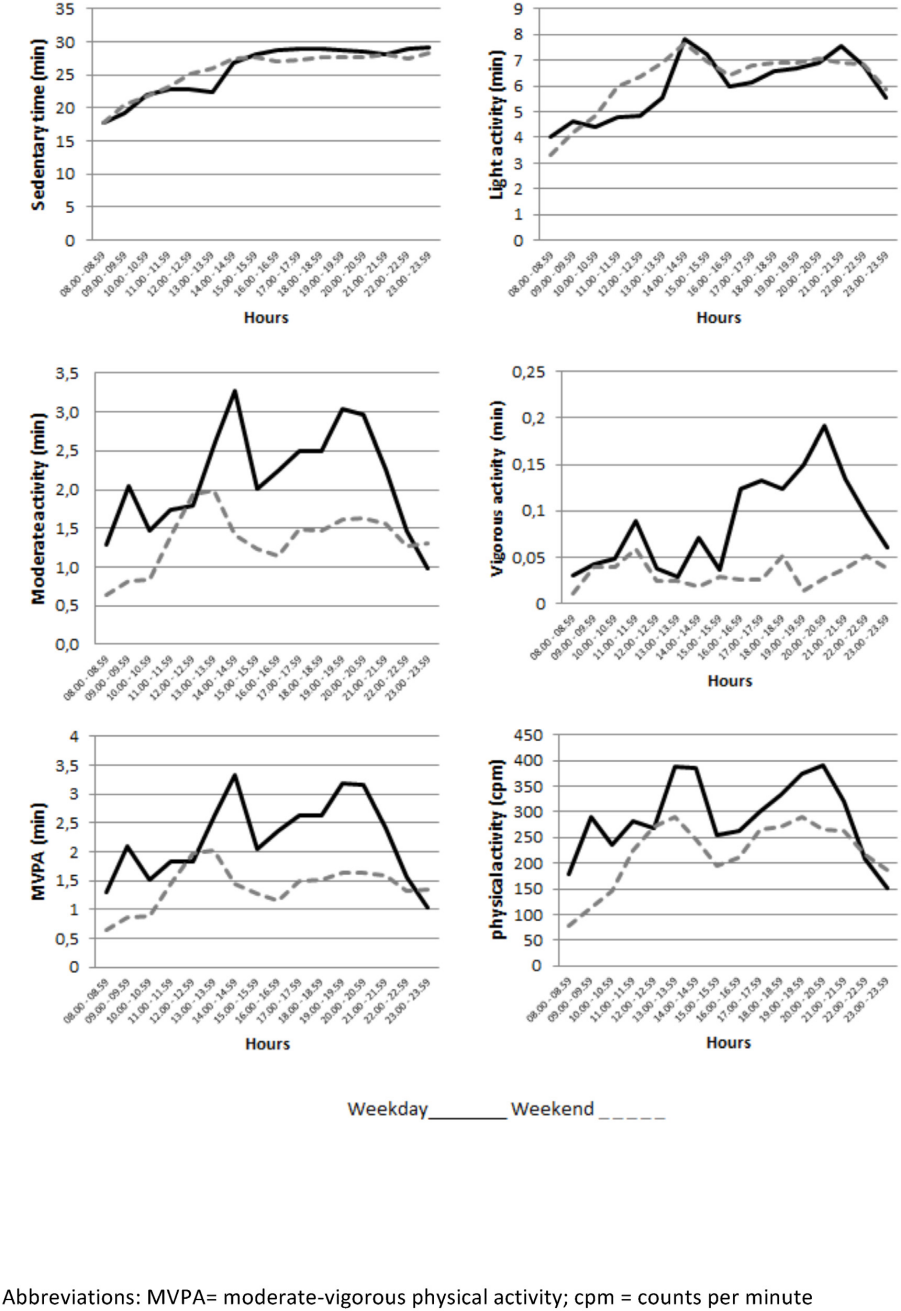
Time spent hourly in sedentary time, light activity, moderate activity, vigorous activity and MVPA and overall PA level (cpm) in Spanish university students on weekdays and weekends.

Fig 2 shows hourly sedentary time, light PA, moderate PA, vigorous PA, MVPA and hourly distribution of overall PA level (cpm). The university students spent more time being sedentary and in light PA during the weekend than they did in the evening. In addition, students spent more time in MVPA and average PA intensity was higher on weekdays. Time spent on MVPA and the average PA intensity was consistently higher from 9.00 to 10.00, 14.00 to 15.00 and 19.00 to 21.00 on weekdays and from 12.00 to 14.00 at the weekend. In addition, throughout the week, MVPA and cpm levels were higher in the afternoon and evening than during the morning.

S1 Fig shows hourly average non-wear time. The average non-wear time was 20.2 minutes on weekdays and 20.3 minutes on weekend days, and the university students were wearing the accelerometer for more time in the evening than during the morning and in the afternoon.

**Fig 3:**
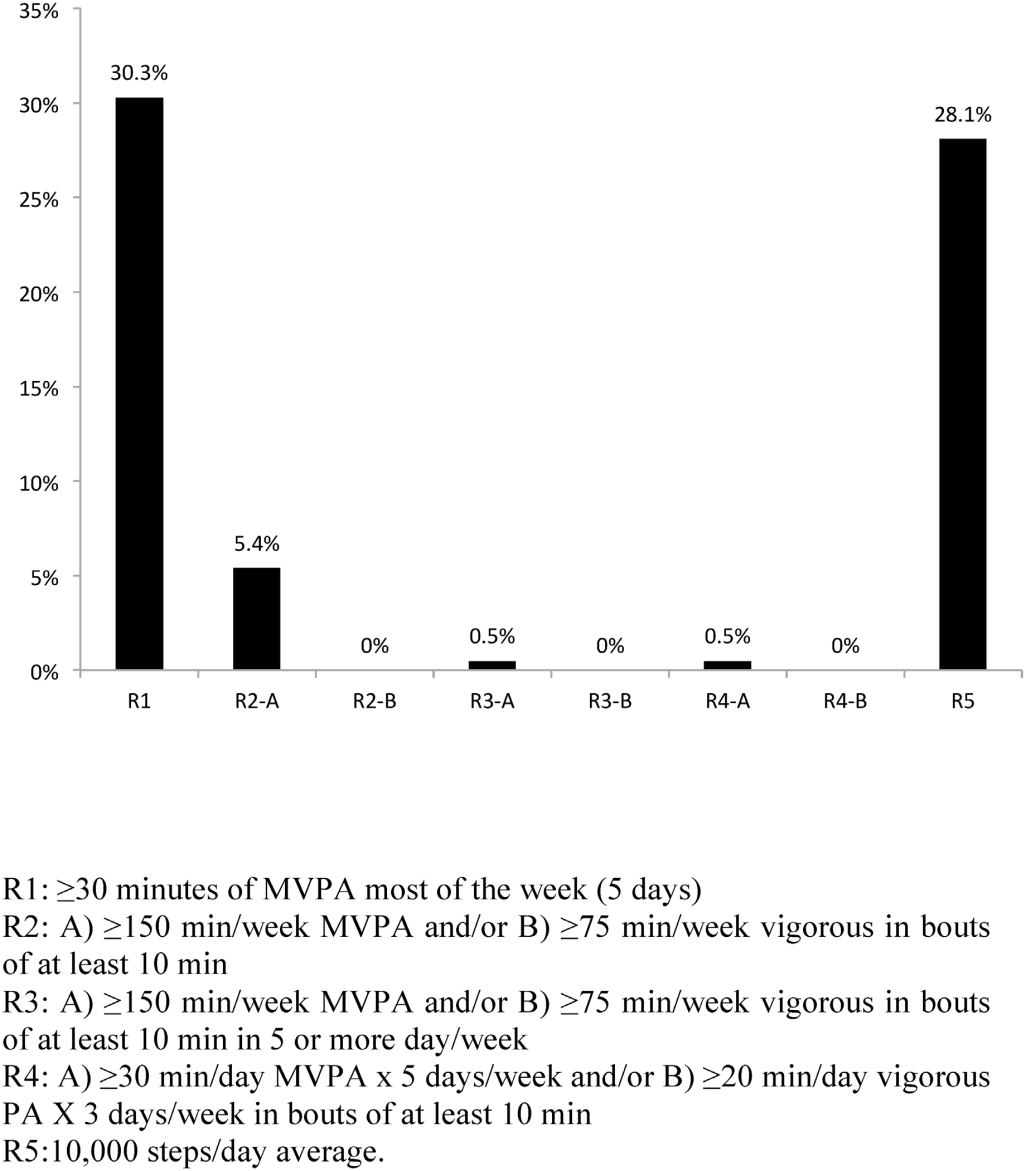
Prevalence (%) of compliance of physical activity recommendations according to the different guidelines.

There were no age, sex or anthropometric differences between students who had five or more valid days and those who did not. The percentage of students meeting the different PA recommendations according to different public health guidelines is presented in Fig 3. R1 was met by 89/294 participants (30.3%), the highest compliance rate of all recommendations. Only 16/296 students (5.4%) met the recommendation of 150 min MVPA/week accumulated in 10 min bouts (R2A) and none of the participants of the total (0/296) accumulated 75 min vigorous PA a week (R2B). When the PA was performed on at least five days, only one person (0.5%) of 212 carried out MVPA (R3A) and, again, none of the 212 fulfilled the recommendations of vigorous PA (R3B). Only one participant (0.5%) of 212 accumulated 30 min of MVPA on at least five days of the week in bouts of at least 10 min (R4A) and none of the 296 fulfilled the recommendations of vigorous PA (R4B). Finally, 55/196 students (28.1%) achieved 10,000 steps/day on average (R5).

## Discussion

There is a lack of studies examining the objectively measured PA patterns in Spanish university students, probably because of the difficulties in getting access to this population; they are a very specific and busy population during the weekdays due to a stable timetable and during periods of exams students spend most of their time studying. Moreover, few studies assess compliance with the recommendations for PA included in the different public health guidelines for adults [4,12,15,28]. Our data reveal that the amount of PA that Spanish university students perform is low, and that the compliance with recommendations for PA depends on the requirements of each of the public health guidelines.

### Physical activity levels and patterns

#### Overall physical activity

To our knowledge, few studies have quantified the levels and patterns of PA in this population in Spain [37–40] and none have measured it objectively. The average of steps/day (12,219) and daily cpm in our study (390) are similar to that reported in other studies, although participants in those studies were older [3,6,41,42]. It is plausible to think that these low values may be due to greater academic demands. During their college years, many students become independent of their families and as a consequence they might spend more time doing chores that involve more housework. And from our point of view, they could cease their physical activity habits. In contrast to what happens in similar studies, where males are more physically active than females [13,43], in our study the levels of PA and steps were similar in men and women. This may be because our sample included university students who have a stable timetable set by their academic classes that cause PA patterns to be similar during this period. In line with our results, Hagstromer et al.[3] did not find sex differences in the Swedish population aged 18 to 39 years, the age range that included our subjects. This study also measured PA objectively and inclusion criteria were similar to ours [3]. Similarly, in the Behrens study there were no significant differences in steps during the seven-day period by gender [44].

#### Time in intensity categories

The mean time spent in sedentary time on weekdays was 594 min/day and at weekends was 618 min/day. These values are higher than those found in the literature [5,6,8,42]. A possible explanation for this is the fact that university students spend many hours sitting in class or studying. Light PA (median 253 min/weekday and 256 min/weekend day) in this study was similar to other studies [4,42], however, in a study of the Portuguese population [5]the number of min/day is lower for those aged between 18 and 29 years, even considering that the cut-offs to calculate this intensity are broader (100–2019 cpm) than those used in our study (100–1951 cpm). Moderate PA was lower (~29 min/day) in our study compared with others [5,6,8,15,42,44] and an alarming result was the number of minutes spent in vigorous PA that was almost nil. These data are truly worrying as consistent evidence argues that the practice of vigorous PA is associated with low cardiovascular and metabolic risk and high aerobic capacity in young adults [33]. Finally, the performed steps number was similar to other studies. [15,28].

#### Physical activity on weekdays vs. weekend days

University students were more active on weekdays than weekends, which is consistent with other similar studies [41,44]. They spent more time on MVPA and walked a higher number of steps on weekdays than on weekends (39.4 vs 22.5 min/day, 9081 vs 7971 steps/day median) and spent less time being sedentary on weekdays than weekends (595.2 vs 618 min/day).

In the current study, on one hand, we found that the participants spent more time being sedentary in the evenings on both weekdays and weekends. On the other, the major peaks of MVPA during weekdays were found to be in the early and late morning and evening. This was probably caused by the activity that happened from home to university, usually done by active commuting, as well as performing sports and leisure activities in the evenings. These data are similar to the data found in a study of objectively measured PA in Hong Kong adults [41]where cpm increased on weekdays during 08.00–08.59, 13.00–13.59 and 18.00–18.59. Probably, the fact that these peaks in PA intensity occur an hour ahead of us, may be due to time differences in each country or culture.

Throughout the weekdays, MVPA and cpm levels were higher in the afternoon and the evening. This differs from the results found in the literature [45], where higher levels of MVPA are in the morning. In addition, it is difficult to compare the samples, because the age group splits our population into two separate populations, those aged 15 to 19 years and adults aged from 20 to 79 years.

### Compliance with different physical activity recommendations

Our results showed, in keeping with other similar studies [3,4,13,46], that young adults do not meet the official PA recommendations for health. In addition, when PA guidelines include more flexible criteria [18], the compliance increases markedly [15]. Previous studies have also shown that low levels of PA are especially evident when bouts of 10 min are considered [28]. If we compare R1 with R4A, we observe these have the same criteria, but without the bouts requirement, and compliance decreases from 30.3% to 0.5%. This indicates that university students accumulate PA during periods of very short duration and/or with frequent interruptions. A study of the Portuguese population [5] also showed that when blocks of 10 min of continuous activity of moderate or vigorous intensity were considered, the prevalence of the active population dramatically decreased, varying from ~70% of people aged 18–29 to ~5% accumulating at least 30 min of PA a day. However, this decrease was much lower in persons between 40 and 64 years as they showed a greater predominance of more continuous activities in leisure time as opposed to intermittent activities, probably more dominant in younger ages. Although there are limited epidemiologic studies that have examined the association with health outcomes between accumulation of PA in 10-minute bouts and without bouts, this encouraging evidence indicates that short bouts of exercise can increase cardiorespiratory fitness, promote weight loss, and improve various biologic health outcomes [47,48]. However, a study of 3,250 adults aged 18 years or older [49]affirmed that bouts of PA lasting > or = 10 min may be a more time-efficient strategy to decrease BMI and waist circumference.

The same thing occurred when we considered activity on less than five days/week, where university students performed 150 minutes of MVPA in bouts of 10 min. The compliance decreased from 5.4% (R2A) to 0.5% (R3A). Scheers et al. [15]showed that 94.2% of men and 86.5% women accumulated ≥150 min MVPA per week, but only 73.8% and 55.7% did so in five or more sessions of ≥30 min/day. Although prevalence in compliance with the recommendations is much higher than in our study, this reflects that removing the frequency and duration requirements resulted in a considerable increase in the prevalence of sufficient MVPA. This fact deserves reflection, for that reason, we know that for a habit to occur, the behaviour needs to be repeated regularly, so strategies must be created for university students not only to increase their PA duration, but also the number of days a week when it is performed.

The percentage of adults who achieved at least 60 or 75 min of vigorous PA per week was zero, with or without the bouts criteria, showing that university students do not perform vigorous PA. Studies in healthy people and in individuals with coronary artery disease suggest that vigorous PA, measured subjectively, provides greater benefits than moderate PA [50,51], so it would be necessary to establish strategies to increase the practice of vigorous PA during this stage. A relevant strategy would be to alert university students that the time taken to implement the recommendations of vigorous-intensity PA is lower than with moderate intensity, so they would not need to spend much time weekly. These results should be taken with caution as PA recommendations are based on epidemiological data that is collected using self-report measures [18] and cannot be directly translated to data using objective methods.

The compliance to get the daily average 10,000 steps was 28.1% (R5). Our results were much lower than a study in South Africa [53], which showed that 77.9% men and 59.4% women met the guidelines. Moreover, the degree of compliance with PA guidelines in university students was also very low, as was that in other studies conducted in Spanish adults [24,26], although these studies used questionnaires to gather their data.

### Limitations

Some limitations should be recognized. First of all, it is possible that accelerometers may produce some reactivity by the participants (Hawthorne effect) in wearing the device; however, unlike self-reports, accelerometer estimates do not suffer from bias due to social desirability and recall problems. In addition, we reduced this effect since the data recorded on the first day of data collection was not considered. Secondly, PA levels and the degree of compliance with the recommendations may be influenced by uncontrolled factors in our study, such as socioeconomic level or weight status. Thirdly, we do not have any reliability data for the people conducting the measures. Fourthly, we are aware that the definition of accelerometer non-wear time could change average wearing time, average accelerometer counts and also might underestimate sedentary time [52]. Thus, our results might have been different if we had used a different definition of accelerometer non-wear time, because when we increase the time period of consecutive zero for exclusion (from 20 to 40 to 60 minutes) total wearing time increases. Note that probably excluding 10 minutes of consecutive zero values as non-wear time might have reduced the estimates of valid wear time for each measurement day, and as consequence would have reduced the number of days that meet the criterion of five or more valid days, underestimating the overall sample size. However, there were no age, sex or anthropometric variable differences between students who had five or more valid days and those who did not, so we think that the representativeness of the sample was guaranteed. Fifth, the lack of agreement between statements for minimum data of accelerometer recordings and recommendations for PA in most guidelines could affect our results, since while it is usually accepted that 4 days of accelerometer data recording is enough to measure PA behaviour [34], accumulating at least 150 minutes of moderate-to-vigorous PA in five or more days a week is recommended in most international guidelines. In order to overcome these limitations, a recent publication stressed the need for technological advances in devices. These advances will allow the accelerometer to be worn on the wrist to increase adherence, to be programmed in the 5 hertz (Hz) to 100 Hz range to better record all kinds of movements and with an interval of recorded time (epochs) of 1 second, so that the activity time will grow exponentially [53]. Finally, accelerometers that had been combined with other sensors (measuring physiological responses) would have guaranteed a more accurate PA measurement.

## Conclusions

Our study shows very low PA levels in both young men and women. The time spent being physically sedentary was higher than others have found in the literature and the number of minutes spent on vigorous PA was almost nil. Total PA levels were significantly higher during the weekdays than the weekend. University students in the province of Cuenca do not comply with international PA recommendations for this population at present, but we note that if using flexible criteria, the compliance with more demanding recommendations increases significantly.

Since this population group is starting adulthood they may not yet have firmly defined their lifestyles. Therefore, strategies that promote an active lifestyle are the key to improve health outcomes and reduce the risk of future disease.

## Supporting Information

S1 Fig Hourly average non-wear time. (TIFF)
