# Supplementary figures and images for: Levels and Patterns of Objectively Assessed Physical Activity and Compliance with Different Public Health Guidelines in University Students
Source: PLoS One. 2015 Nov 4;10(11):e0141977. doi: 10.1371/journal.pone.0141977 (PMC4633238; doi:10.1371/journal.pone.0141977)

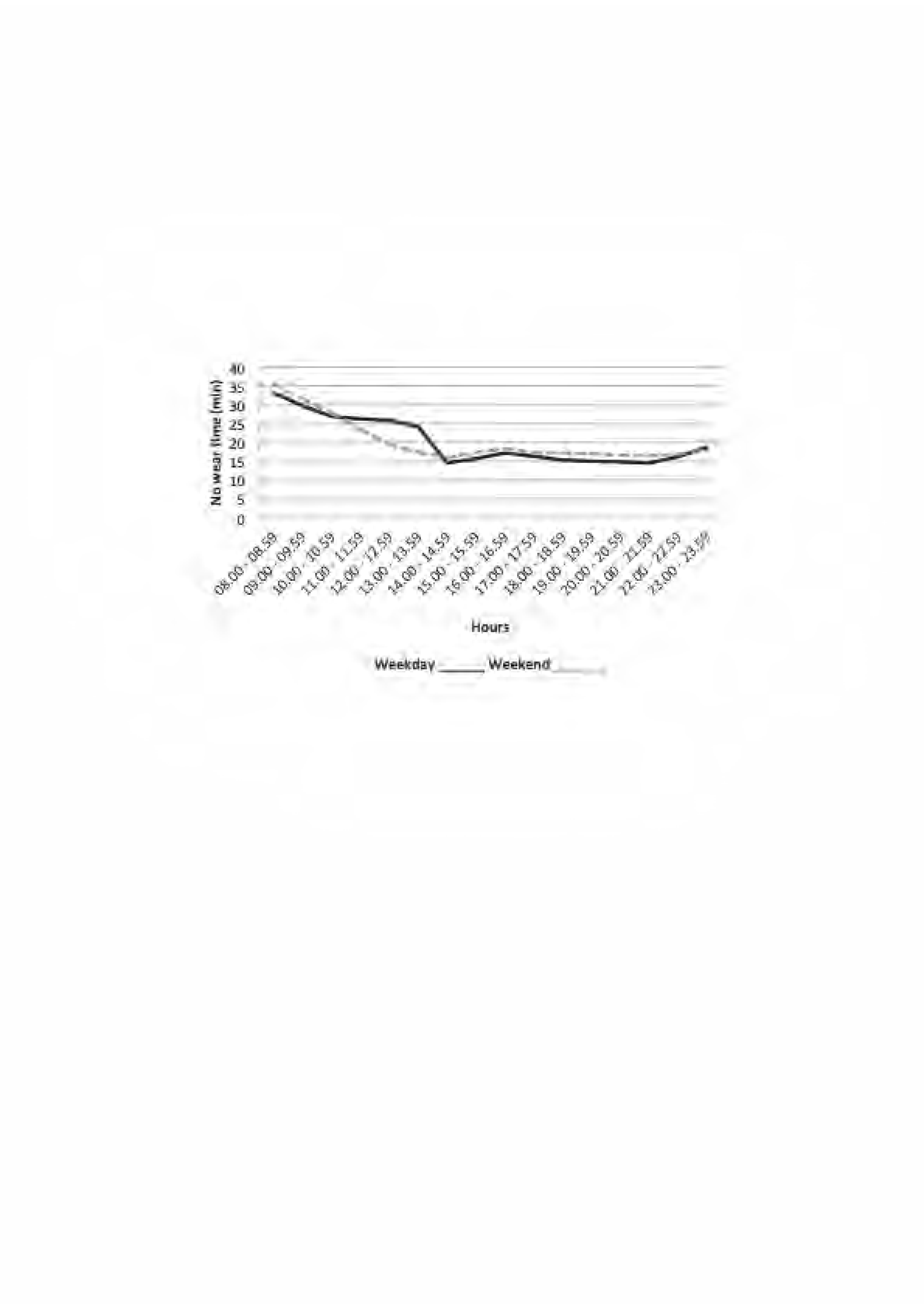

Supplement: S1 Fig — (TIFF) [file pone.0141977.s001.tiff]
